# Short-course radiotherapy for rectal cancer: real-world evidence in Argentina

**DOI:** 10.3332/ecancer.2023.1555

**Published:** 2023-06-01

**Authors:** Natalia S Tissera, Berenice Freile, Federico Waisberg, Federico Esteso, Mariana Galli, Fernando Sanchez Loria, Romina Luca, Ivana Inés Pedraza, Diego Hernán Enrico, Carolina Chacón, Eduardo Huertas, Matías Rodrigo Chacón, Juan Manuel O’Connor

**Affiliations:** 1 Upper Gastrointestinal Cancer Translational Research Group, Vall´d Hebron Institute of Oncology (VHIO), 08035 Barcelona, Spain; 2 Department of Oncology, Alexander Fleming Institute, CABA C1426, Argentina; 3 Department of Radiotherapy, Alexander Fleming Institute, CABA C1426, Argentina; 4 Department of Surgery, Alexander Fleming Institute, CABA C1426, Argentina; a https://orcid.org/0000-0002-3396-6878; b https://orcid.org/0000-0003-3192-126X; c https://orcid.org/0000-0003-4435-5068; d https://orcid.org/0000-0003-1977-9846; e https://orcid.org/0000-0003-0147-2192; f https://orcid.org/0000-0001-9708-0649; g https://orcid.org/0000-0001-8564-905X; h https://orcid.org/0000-0001-9679-8368; i https://orcid.org/0000-0003-4121-6855; j https://orcid.org/0009-0004-8556-6325; k https://orcid.org/0000-0003-3473-0928; l https://orcid.org/0000-0001-6872-4185; m https://orcid.org/0000-0002-6975-5466

**Keywords:** short-course radiotherapy, rectal cancer, watch and wait, clinical complete response, Latin America

## Abstract

**Background:**

Short-course radiotherapy (SCRT) of 25 Gy in five daily fractions is a recommended strategy in the neoadjuvant setting for resectable locally advanced rectal cancer (LARC), as well as in cases of metastatic disease for local control. There is scarce information regarding the use of SCRT for patients who have received nonoperative management.

**Objectives:**

To describe the characteristics of patients who received treatment with SCRT for LARC and metastatic rectal cancer, toxicity, and the approach after radiation treatment.

**Methods:**

This is a retrospective analysis of all patients who underwent SCRT for rectal cancer at the Alexander Fleming Institute from March 2014 to June 2022.

**Results:**

In total, 44 patients were treated with SCRT. The majority were male (29, 66%), with a median age of 59 years (interquartile range 46–73). Most patients had stage IV disease (26, 59.1%), followed by LARC (18, 40.9%). Most lesions were located in the middle rectum (30, 68%). The majority of LARC patients underwent SCRT followed by consolidation chemotherapy (ChT) (16/18, 89%), while most patients with metastatic disease underwent SCRT followed by consolidation ChT (14/26, 53.8%). A clinical complete response (cCR) was documented in 8/44, 18.2% of patients. Most patients with LARC and cCR were managed by a watch and wait approach (5/18, 27.7%). Local recurrence was observed in LARC cases (2/18, 11.1%). Patients who underwent SCRT following consolidation ChT were more likely to have adverse events (AEs) than those undergoing induction ChT following SCRT (11/30, 36.7% versus 3/12, 25%, *p* = 0.02)

**Conclusion:**

In a subgroup of patients diagnosed with LARC and treated with SCRT followed by ChT, surgical treatment could be omitted after they achieved a cCR. Local recurrence was similar to that reported in a previous study. SCRT is a reasonable option for local disease control in stage IV disease, yielding low toxicity rates. Therefore, decisions must be made by a multidisciplinary team. Prospective studies are necessary to reach further conclusions.

## Introduction

Short-course radiotherapy (SCRT), which allows the delivery of 25 Gy in five daily fractions, has emerged as an attractive strategy for rectal cancer treatment [1]. Surgery can safely be deferred after SCRT, allowing an opportunity to deliver chemotherapy (ChT) preoperatively rather than postoperatively [2, 3]. In cases of metastatic disease, this represents an effective treatment option to improve local control and avoid colostomy in a subset of patients [4].

The randomised open-label phase 3 Rectal cancer And Preoperative Induction therapy followed by Dedicated Operation (RAPIDO) trial determined that SCRT followed by ChT has a higher pathologic complete response rate than long-course chemoradiotherapy (LCCRT), as well as less disease-related treatment failure and higher distant metastasis-free survival [5, 6]. Moreover, SCRT offers a shorter overall treatment duration, low toxicity rates, and good quality of life (QoL), and it is highly cost-effective [7, 8].

The incidence of clinical complete response (cCR) with SCRT is the key endpoint to define nonoperative management in lower rectal cancer. In a prospective study (*n* = 19) that explored SCRT followed by 16 weeks of 5-fluorouracil, folinic acid, and oxaliplatin (FOLFOX) or capecitabine and oxaliplatin (CAPOX), 74% cCR and 57% (95% CI 36%–91%) organ preservation at 2 years were reported [9]. In addition, a retrospective study of SCRT followed by 8 weeks of FOLFOX reported a 47% cCR [10]. Another retrospective study compared SCRT, SCRT plus ChT, and LCCRT. The clinical response rates were 8%, 27% and 18%, respectively [11].

A meta-analysis showed that SCRT and LCCRT as neoadjuvant treatments for locally advanced rectal cancer (LARC) have an adequate safety profile without significant differences regarding severe acute or late toxicities [12].

Here, we report the first retrospective data in Argentina describing the characteristics of patients treated with SCRT for LARC and metastatic rectal cancer, toxicity and subsequent therapeutic decisions after radiation treatment.

## Methods

This is a retrospective analysis of all patients undergoing SCRT for rectal cancer at the Alexander Fleming Institute between March 2014 and June 2022. Descriptive statistics were used to summarise variables, including medians and interquartile ranges (IQR). Fisher’s test was carried out to compare toxicities. The inverse of Kaplan‒Meier method was used to calculate follow-up. Disease-free survival (DFS) was calculated from the date of cCR (obtained by endoscopy, digital rectal examination (DRE) and pelvic magnetic resonance imaging (MRI) until recurrence). Follow-up was censored considering the last patient visit.

Toxicities were reported by the Common Terminology Criteria for Adverse Events (CTCAE v5.0) [13]. European Society for Medical Oncology (ESMO) guidelines were used for the risk group classification of LARC [14]. Early category included tumours defined as cT1–cT2, cT3a/b if medium or high, N0 (or also cN1 if high), clear mesorectal fascia (MRF) and no extramural venous invasion (EMVI). The intermediate category included tumours cT2 very low, cT3 MRF- (unless cT3a(b) and mid- or high rectum, N1-2, EMVI+, limited cT4aN0. Advanced LARC included cT3 tumours with any MRF involved, cT4a/b lesions, and all patients who had lateral lymph node involvement. Statistical analyses were performed using IBM Statistical Package for the Social Sciences Statistics 29.0.

## Results

Between March 2014 and June 2022, 44 patients were treated with SCRT. The majority were male (29/44, 66%), and the median age was 59 years (IQR 46–73). Most patients (26/44, 59.1%) had stage IV disease, followed by clinical stages II–III (18/44, 40.9%).

According to MRI, most lesions were localised in the middle rectum (30/44, 68%) and the lower rectum (14/44, 32%). According to the ESMO risk classification, LARC tumours were classified as early (2/18, 11%), intermediate (5/18, 28%), and advanced (11/18, 61%). Molecular testing was performed mainly in metastatic disease. Deficient mismatch repair (dMMR) was found in metastatic disease (2/30, 6.6%), and Kirsten rat sarcoma virus (KRAS) mutations were evidenced in 12/20, 60% of the evaluated samples. Patient characteristics are summarised in Table 1.

**Figure 1.**
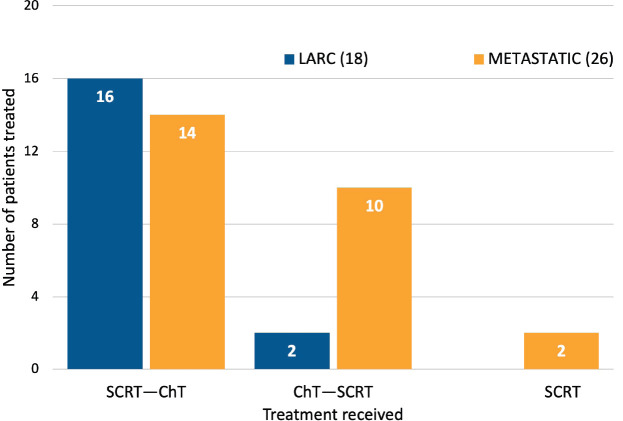
Treatment received.

Patients with LARC (16/18, 89%) underwent SCRT followed by consolidation ChT and induction ChT followed by SCRT (2/18, 11%). Among the subgroup with metastatic disease, most patients underwent SCRT followed by consolidation ChT (14/26, 53.8%). Other strategies comprised induction ChT followed by SCRT (10/26, 38.5%) and SCRT alone (2/26, 7.7%) (Figure 1). The most common regimens used in consolidation ChT were CAPOX (capecitabine 1,000 mg/m^2^ orally twice daily on days 1–14, oxaliplatin 130 mg/m^2^ intravenously on day 1 and a ChT-free interval between days 15 and 21) (13, 43.3%) and CAPOX plus bevacizumab (7.5 mg/kg day 1 every 3 weeks) when induction therapy was recommended (5, 42%). The modality of SCRT most commonly used was 3D (39/44 88.6%), followed by intensity-modulated radiotherapy (IMRT) (5/44, 11.3%).

**Figure 2.**
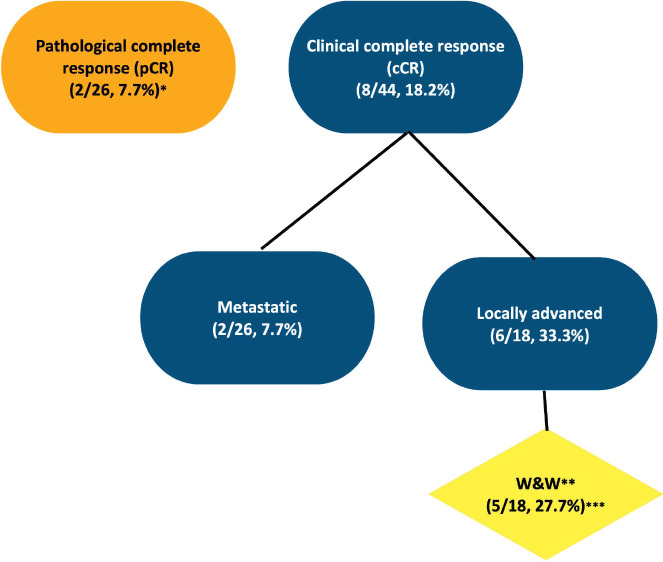
Clinical and pathological response.

Among the patients who underwent surgery, most had metastatic disease (17/29, 58.6%). With a median follow-up of 20 months, cCR was evidenced in 8/44, 33% of cases (6/18, 33% of patients with LARC, and 2/26, 7.7% of cases with metastatic disease). Importantly, 5/18, 27.7% patients with LARC and cCR were managed by a watch-and-wait (*w*&*w*) approach (Figure 2).

The local recurrence rate of LARC cases occurred in 2/18, 11.1% cases. One of them was a patient under the *w*&*w* strategy who exhibited a relapse occurring after 14 months. This patient underwent surgery and is currently disease-free.

**Figure 3.**
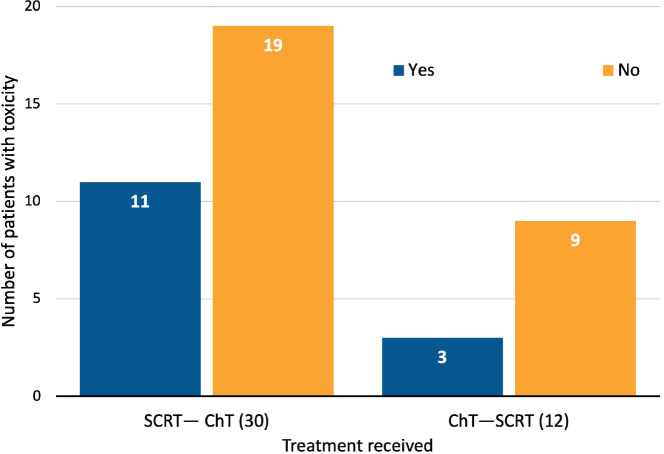
Acute toxicity (G1-2) during SCRT (CTCAE v5.0).

Those patients who underwent SCRT followed by consolidation ChT were more likely to present adverse events (AEs) than patients who had induction ChT followed by SCRT (11/30, 36.7% versus 3/12, 25%, *p* = 0.02) (Figure 3). Acute toxicities (G1–G2) during SCRT reported in this cohort included proctitis (9), diarrhea (3), mucositis (3), dermatitis (1), cystitis (1), and neuropathy (1). Neither G3 nor higher AEs or treatment discontinuations were reported (Table 2).

## Discussion

Rectal cancer is associated with challenging therapeutic decisions. Different approaches need to be carefully analyzed considering that treatment protocols may include modalities associated with impairing toxicities and deterioration of QoL. For this reason, therapeutic strategies associated with a high tumour response and low occurrence of related AEs are largely needed.

To the best of our knowledge, there are only three prospective trials that have explored the effectivity of SCRT followed by ChT. Kim *et al* [9] designed a nonrandomised trial that enrolled 20 patients with LARC to receive SCRT followed by ChT (FOLFOX or CAPOX). Patients were assessed for a clinical response 3–8 weeks after ChT completion with pelvic MRI, endoscopy and DRE. For the primary endpoint, 13/19 (68%) patients maintained cCR at 1 year after SCRT. Patients with cCR were associated with improved 2-year disease-free survival (93% versus 67%; *p* = 0.006), distant metastasis-free survival (100% versus 67%; *p* = 0.03) and overall survival (100% versus 67%; *p* = 0.03). Four of 12 patients (33%) initially deemed to require surgery and permanent stoma underwent nonoperative management, and 3 of 12 (25%) were able to undergo low anterior resection. Grade 3 or 4 acute toxicities were observed during SCRT (10, 53%), and during ChT, neutropenia and febrile neutropenia were the most frequent AEs. Importantly, an anorectal function was assessed by the Functional Assessment of Cancer Therapy-Colorectal cancer score, showing that anorectal function did not differ from baseline to the 1-year measure. Therefore, the researchers showed that SCRT-ChT seems to preserve both organ and anal function [9].

Two other prospective studies explored this question. One of the studies included 66 patients and assessed the effectivity of the *w*&*w* approach in elderly patients (70 years) with small cancers (tumour length of 5 cm and circumferential extent of 60%). The primary endpoint was the rate of regrowth rate in the patient’s subgroup who achieved cCR and received *w*&*w* management. The other investigation was a multi-institutional study that included 424 patients and assessed the *w*&*w* approach in patients receiving routine LCCRT. In a polled analysis that included both studies, a total of 490 patients were evaluated. SCRT, SCRT plus ChT, and LCCRT were received by 41%, 40% and 19% of patients, respectively. Importantly, SCRT was most often administered to elderly patients or patients with small cancers, whereas SCRT plus ChT was given in cases of advanced disease. In total, 73 (15%) patients achieved cCR. In 51% of these individuals, the diagnosis was made at the first assessment. Of the 73 patients who achieved cCR, 71 received *w*&*w* management. After 3 years, the regrowth was comparable, around 35%–40% in all three subgroups. Toxicities were not reported [15].

Our cohort of 44 patients with rectal cancer reported a total cCR of 18.2%, meaning 33.3% of patients had LARC. Notably, this subgroup was characterised as having high-risk features, such as T3 tumours, lymph nodes or Circumferential resection margin (CRM) involvement. Our result is similar to those of other published series (between 35% and 79%). However, it is lower than the reported cCR rates of patients undergoing LCCRT, which are approximately 50% [6, 10, 16]. For the study assessments, we used the diagnosis of cCR as defined by Habr-Gama *et al* [17] by DRE, endoscopy, and MRI at 34–38 weeks after treatment initiation [18, 19]. On the other hand, current guidelines also recommend that in patients with near complete response (CR) or major tumour shrinkage, surveillance should be conducted for 4–8 weeks before making a final decision on local surgical treatment [18].

Moreover, the results of long-term local recurrence with SCRT are a matter of concern. Local recurrence after initial cCR usually occurs in the first 2–3 years of treatment; thus, a 3-year follow-up period is strongly recommended for early detection [18]. In the RAPIDO trial, 5-year local recurrence rate was reported more frequently after SCRT (10% versus 6%) of patients, while the reduction in disease-related treatment failure and distant metastases was maintained after 5 years. In addition, the study also found a higher local recurrence in those patients of the experimental group treated with the 3D technique (12% versus 6%), while no such difference was observed in patients treated with IMRT (6% versus 5% respectively). While the irradiated volumes with either technique should not differ, it is possible that in some centers individual volume delineation is not routinely used for 3D-CRT as opposed to IMRT. However, this should not affect the coverage of the anastomotic region, usually located centrally in the target volume, which was the main Locoregional recurrence (LRR) localisation in the experimental arm of the RAPIDO trial. This excess of LRR may be more related to the mesorrectum being more often breached in the experimental arm [20]. In contrast, authors of the Organ preservation in rectal adenocarcinoma (OPRA) trial study reported recurrence rates of 40% and 27% in patients who received induction ChT followed by LCCRT and LCCRT followed by consolidation ChT, respectively [21]. However, in our series, the local relapse rate in the LARC subgroup was 11.1%. One of these patients had received *w*&*w* management and, after local relapse, underwent surgery and is disease free to date. These observations raise the question of whether deferring surgery predisposes some patients to distant metastases and indicate the need for better risk stratification for nonsurgical treatment.

SCRT has shown an adequate safety profile compared to conventional LCCRT. The most common acute toxicities are gastrointestinal, with enteritis being the most frequent, followed by urinary symptoms [12]. Nevertheless, SCRT is associated with a variety of long-term effects compared to LCCRT [22]. The most frequent late toxicities are grade 2 gastrointestinal AEs such as chronic diarrhea, ileus, bowel obstruction, or perforation, followed by urinary AEs such as frequency, urgency or hydronephrosis. In our study, only G1–2 acute toxicities, especially gastrointestinal AEs, were reported. However, the follow-up of our series was too short to assess late toxicities.

Whereas we noted that radiation therapy is a cornerstone of rectal cancer treatment, it is important to assess how the side effects affect patients’ QoL. Large systematic reviews and long-term follow-up clinical trials have shown that radiotherapy (RT) toxicities, particularly fecal incontinence and sexual dysfunction, affect QoL. Many QoL questionnaires for rectal cancer have been validated, and recent studies have included patient-reported outcome measures and validated QoL metrics to better inform providers and patients. Information about QoL is disparate. While most studies do not show a difference in QoL between SCRT and LCCRT [23–25], a population-level study in the UK of patients with rectal cancer investigated functional outcomes and health-related QoL 12–36 months after curative rectal cancer treatment using survey of patient-reported outcomes and administrative data. Surveys were returned by 6.713/10.452 patients. They observed that patients who received preoperative radiotherapy reported clinically and statistically significantly worse bowel control (43.6% versus 33.0%; OR = 1.55, 95% CI 1.26–1.91), severe urinary leakage (7.2% versus 3.5%; OR = 1.69, 95% CI 1.18–2.43) and severe sexual difficulties (34.4% versus 18.3%; OR = 1.73, 95% CI 1.43–2.11) compared to patients who had surgery alone. Patients who received SCRT reported worse bowel control than those who had LCCRT. Patients with a stoma reported more sexual difficulties and worse Health-related quality of life (HRQL) outcomes [26].

Regarding the role of SCRT in the metastatic stage, our institute prioritises the use of SCRT over the standard regimen, as it allows avoiding colostomy, decreasing pain, bleeding and mass effect in many cases with low toxicity. However, the recommendations are based on a limited level of evidence [27]. More randomised comparative trials are needed to determine the benefits compared with other RT protocols or with different palliative treatments (i.e., stenting or bypass surgery).

In summary, since the reporting of data from the RAPIDO trial and, most notably, during the COVID-19 pandemic lockdown, when it was critical to shorten radiation regimens and delay surgeries, neoadjuvant SCRT (or SCRT followed by ChT) and nonsurgical treatment of rectal adenocarcinoma has become an attractive approach. In many countries, neoadjuvant SCRT followed by ChT has become the standard preoperative strategy for operable rectal adenocarcinomas based on long-term outcomes comparable to those of LCCRT in terms of efficacy and QoL. In light of the aforementioned findings, SCRT has become our standard therapy for patients with localised or LARC (low-rectum localisation of disease), for patients who are thought to be unable to tolerate LCCRT, and for patients with metastatic disease to minimise delays in the onset of systemic therapy and to manage symptoms. In patients who achieve a cCR, nonoperative management may be offered. We strongly recommend a multidisciplinary team in which all decisions are discussed. On the other hand, we emphasise the importance of being aware of the microsatellite instability status before making a treatment decision, as a published study with immunotherapy have shown that such patients may avoid radiotherapy and surgery after treatment with checkpoint immune inhibitors [28].

## Conclusion

Current studies on the *w*&*w* approach are based on LCCRT, and there are scarce data on SCRT and nonsurgical management. To our knowledge, this is the first retrospective study on patients treated with SCRT and ChT in Argentina. A proportion of patients who were diagnosed with LARC achieved cCR without surgery with mild toxicity. In addition, SCRT allowed favorable local control rates in patients with stage IV disease. Despite the limitations of a retrospective study, this is the only evidence available in Latin America that shows the preliminary safety and efficacy of SCRT followed by ChT reported in the literature, a treatment paradigm that is increasingly implemented worldwide. Studies evaluating the role of biomarkers and circulating tumour DNA to predict and prognosticate outcomes in patients who do not undergo surgery are needed. Prospective studies are warranted to support SCRT followed by ChT as a nonsurgical treatment strategy for rectal cancer. In the meantime, decisions should be made on a case-by-case basis by a multidisciplinary team.

## Conflicts of interest

The authors declare no conflict of interest for this manuscript.

## Funding

None.

## Figures and Tables

**Table 1.** Characteristic of the patients (*n* = 44).

	*n* (%)
Median age at diagnosis	59 (IQR 46–73)
Sex Female Male	15 (34%) 29 (66%)
Stage LARC Metastatic	18 (40.9%) 26 (59.1%)
Localisation Medium Low	30 (68%) 14 (32%)
Histology Adenocarcinoma	44 (100%)
Molecular features dMMR KRAS mutated BRAF mutated	2/30 (6.6%) 12/20 (60%) 0/12 (0%)
Risk classification of LARC^a^ cT1-3a, N0 cT3c/d cN1-2, EMVI+, CRM− cT3 CRM + o cT4a/b, N+	2 (11%) 5 (28%) 11 (61%)
Local recurrence LARC	2/18 (11.1%)

IQR, interquartile range; LARC, locally advanced rectal cancer; dMMR, Deficient mismatch repair; CRM, Circumferential resection margin; EMVI, Extramural Venous invasion; N+, Positive lymph nodes

a Risk classification of rectal cancer by ESMO clinical practice guidelines 2017

**Table 2.** AEs of patients (*N* = 14).

Toxicity	*n*
Proctitis	9 (50%)
Diarrhea	3 (16.6%)
Mucositis	3 (16.6%)
Dermatitis	1 (5.6%)
Cystitis	1 (5.6%)
Neuropathy	1 (5.6%)

## References

[1] Wo JY, Anker CJ, Ashman JB (2021). Radiation therapy for rectal cancer: executive summary of an ASTRO clinical practice guideline. Pract Radiat Oncol.

[2] Radu C, Berglund A, Påhlman L (2008). Short-course preoperative radiotherapy with delayed surgery in rectal cancer - a retrospective study. Radiother Oncol J Eur Soc Ther Radiol Oncol.

[3] Nilsson PJ, van Etten B, Hospers GA (2013). Short-course radiotherapy followed by neo-adjuvant chemotherapy in locally advanced rectal cancer – the RAPIDO trial. BMC Cancer.

[4] Picardi V, Deodato F, Guido A (2016). Palliative short-course radiation therapy in rectal cancer: a phase 2 study. Int J Radiat Oncol.

[5] Skowron KB, Hurst RD, Umanskiy K (2020). Caring for patients with rectal cancer during the COVID-19 pandemic. J Gastrointest Surg.

[6] Bahadoer RR, Dijkstra EA, Etten B (2021). Short-course radiotherapy followed by chemotherapy before total mesorectal excision (TME) versus preoperative chemoradiotherapy, TME, and optional adjuvant chemotherapy in locally advanced rectal cancer (RAPIDO): a randomised, open-label, phase 3 trial. Lancet Oncol.

[7] Chin RI, Otegbeye EE, Kang KH (2022). Cost-effectiveness of total neoadjuvant therapy with short-course radiotherapy for resectable locally advanced rectal cancer. JAMA Netw Open.

[8] Kane C, Glynne-Jones R (2019). Should we favour the use of 5 × 5 preoperative radiation in rectal cancer. Cancer Treat Rev.

[9] Kim H, Pedersen K, Olsen JR (2021). Nonoperative rectal cancer management with short-course radiation followed by chemotherapy: a nonrandomized control trial. Clin Colorectal Cancer.

[10] Jia AY, Narang A, Safar B (2019). Sequential short-course radiation therapy and chemotherapy in the neoadjuvant treatment of rectal adenocarcinoma. Radiat Oncol Lond Engl.

[11] Hammarström K, Imam I, Mezheyeuski A (2020). A comprehensive evaluation of associations between routinely collected staging information and the response to (Chemo) radiotherapy in rectal cancer. Cancers.

[12] Liscu HD, Miron AI, Rusea AR (2021). Short-course radiotherapy versus long-course radio-chemotherapy as neoadjuvant treatment for locally advanced rectal cancer: meta-analysis from a toxicity perspective. Mædica.

[13] US Department of Health and Human Services (2017). Common terminology criteria for adverse events. version 5.0 Published November 27, 2017.

[14] Glynne-Jones R, Wyrwicz L, Tiret E (2017). Rectal cancer: ESMO clinical practice guidelines for diagnosis, treatment and follow-up. Ann Oncol.

[15] Pietrzak L, Cencelewicz A, Rutkowski A (2022). The utility of short-course radiotherapy in a watch-and-wait strategy for rectal cancer - the need to measure the interval to tumour response assessment from the radiation start. Acta Oncol Stockh Swed.

[16] Chin RI, Roy A, Pedersen KS (2022). Clinical complete response in patients with rectal adenocarcinoma treated with short-course radiation therapy and nonoperative management. Int J Radiat Oncol Biol Phys.

[17] Habr-Gama A, Sabbaga J, Gama-Rodrigues J (2013). Watch and wait approach following extended neoadjuvant chemoradiation for distal rectal cancer: are we getting closer to anal cancer management?. Dis Colon Rectum.

[18] Fokas E, Appelt A, Glynne-Jones R (2021). International consensus recommendations on key outcome measures for organ preservation after (chemo) radiotherapy in patients with rectal cancer. Nat Rev Clin Oncol.

[19] Battersby NJ, Dattani M, Rao S (2017). A rectal cancer feasibility study with an embedded phase III trial design assessing magnetic resonance tumour regression grade (mrTRG) as a novel biomarker to stratify management by good and poor response to chemoradiotherapy (TRIGGER): study protocol for a randomised controlled trial. Trials.

[20] Dijkstra EA, Nilsson PJ, Hospers GAP (2023). Locoregional failure during and after short-course radiotherapy followed by chemotherapy and surgery compared to long-course chemoradiotherapy and surgery – a five-year follow-up of the RAPIDO trial. Ann Surg.

[21] Garcia-Aguilar J, Patil S, Gollub MJ (2022). Organ preservation in patients with rectal adenocarcinoma treated with total neoadjuvant therapy. J Clin Oncol.

[22] Bujko K, Nowacki MP, Nasierowska-Guttmejer A (2004). Sphincter preservation following preoperative radiotherapy for rectal cancer: report of a randomised trial comparing short-term radiotherapy vs. conventionally fractionated radiochemotherapy. Radiother Oncol J Eur Soc Ther Radiol Oncol.

[23] Neibart SS, Manne SL, Jabbour SK (2020). Quality of life after radiotherapy for rectal and anal cancer. Curr Colorectal Cancer Rep.

[24] Pietrzak L, Bujko K, Nowacki MP (2007). Quality of life, anorectal and sexual functions after preoperative radiotherapy for rectal cancer: report of a randomised trial. Radiother Oncol J Eur Soc Ther Radiol Oncol.

[25] Couwenberg AM, Burbach JPM, Intven MPW (2019). Health-related quality of life in rectal cancer patients undergoing neoadjuvant chemoradiation with delayed surgery versus short-course radiotherapy with immediate surgery: a propensity score-matched cohort study. Acta Oncol Stockh Swed.

[26] Downing A, Glaser AW, Finan PJ (2019). Functional outcomes and health-related quality of life after curative treatment for rectal cancer: a population-level study in England. Int J Radiat Oncol Biol Phys.

[27] Cameron MG, Kersten C, Vistad I (2014). Palliative pelvic radiotherapy of symptomatic incurable rectal cancer – a systematic review. Acta Oncol.

[28] Cercek A, Lumish M, Sinopoli J (2022). PD-1 blockade in mismatch repair–deficient, locally advanced rectal cancer. N Engl J Med.

